# Diagnostic accuracy and economic impact of three work-up strategies identifying risk groups in endometrial cancer, fully incorporating sentinel lymph node algorithm

**Published:** 2020-10-08

**Authors:** AA Novelli, A Puppo, M Ceccaroni, E Olearo, G Monterossi, G Mantovani, S Pelligra, PL Olearo, F Fanfani, G Scambia

**Affiliations:** Department of Obstetrics and Gynaecology, “Regina Montis Regalis” Hospital, Mondovì (Cuneo), Italy; Università Cattolica del Sacro Cuore, Rome, Italy; Department of Obstetrics and Gynaecology, Santa Croce e Carle Hospital, Cuneo, Italy; Department of Obstetrics and Gynaecology, Gynaecologic Oncology and Minimally-Invasive Pelvic Surgery, International School of Surgical Anatomy, IRCCS Sacro Cuore-Don Calabria Hospital, Negrar (Verona), Italy; Fondazione Policlinico Universitario Agostino Gemelli IRCCS, Rome, Italy

**Keywords:** endometrial cancer, risk groups, lymphadenectomy, surgical staging, work-up strategy, sentinel lymph node

## Abstract

**Background:**

According to the European Society for Medical Oncology/ European Society of Gynaecological Oncology/European Society for Radiotherapy and Oncology (ESMO/ESGO/ESTRO) Consensus Conference, the role of preoperative risk groups (RGs) in endometrial cancer (EC) is to direct surgical nodal staging. We compared diagnostic accuracy and economic impact of three work-up strategies to identify RGs.

**Methods:**

A retrospective multicentre study including patients with early-stage EC. The three different work-up strategies were as follows:

-Mondovì Hospital: transvaginal ultrasonography, pelvic magnetic resonance imaging (MRI); frozen section examination of the uterus in case of imaging discordance. High-risk patients underwent abdominal computed tomography.

-Gemelli Hospital: transvaginal ultrasonography, MRI, One-Step Nucleic Acid Amplification (OSNA) of sentinel lymph node (SLN); frozen section examination of the uterus in case of imaging discordance.

-Negrar Hospital: positron emission tomography (PET), frozen section examination of the uterus and of SLN. For statistical purposes patients were assigned, preoperatively and postoperatively, to two groups: group A (high-risk) and group B (not high-risk).

**Results:**

Three hundred eighty-five patients were included (93 Mondovì, 215 Gemelli, 77 Negrar). Endometrial biopsy errors led to 47.3% misclassifications. Test accuracy of Mondovì, Gemelli and Negrar strategies was 0.83 (95%CI 0.734-0.901), 0.95 (95%CI 0.909-0.975) and 0.94 (95%CI 0.866-0.985), respectively. Preoperative work-up mean cost per patient in group A was €514.5 at Mondovì, €868.5 at Gemelli, and €1212.8 at Negrar hospital (p-value < 0.001), while in group B was €378.8 at Mondovì, €941.2 at Gemelli, and €1848.4 at Negrar hospital (p-value < 0.001).

**Conclusions:**

In our study, work-up strategies with more relevant economic impact showed a better diagnostic accuracy. Upcoming guidelines should specify recommendations about the gold standard work-up strategy, including the role of SLN.

## Introduction

To date, the European recommendations regarding the management of endometrial cancer are provided by the European Society for Medical Oncology (ESMO), the European Society of Gynaecological Oncology (ESGO), and the European Society for Radiotherapy and Oncology (ESTRO) Consensus Conference (Colombo et al., 2016; Colombo et al., 2017). In this consensus conference early-stage endometrial cancer (EC) patients were classified in three preoperative risk groups (low, intermediate and high risk); their role is to direct surgical nodal staging. The same consensus conference described a wide spectrum of work-up tools to identify preoperative risk groups, allowing hospitals to adopt different work-up strategies in clinical practice; at least one of expert ultrasound, magnetic resonance imaging (MRI) or intraoperative pathological examination of the uterus to assess myometrial invasion, and other imaging methods, such as computed tomography (CT), MRI, positron emission tomography (PET), PET-CT or expert ultrasound to assess ovarian, nodal, peritoneal or metastatic disease (Colombo et al., 2016). Lymph node metastasis is the most important prognostic factor in early-stage EC, while the therapeutic role of systematic pelvic and para- aortic lymphadenectomy is still debated (Benedetti Panici et al., 2008; Kitchener et al., 2009; Todo et al., 2010; Bogani et al., 2014). Sentinel lymph node (SLN) biopsy represents a reasonable alternative to lymphadenectomy for nodal assessment in low risk and intermediate risk patients (Ballester et al., 2011; Holloway et al., 2017) and has been integrated in National Comprehensive Care Network guidelines (NCCN 2020). There is increasing evidence about SLN biopsy as being an accurate and safe nodal staging tool also for the high risk group (Ehrisman et al., 2016; Soliman et al., 2017; Touhami et al., 2017).

Since the ESMO/ESGO/ESTRO Consensus Conference considers preoperative risk groups to be of paramount importance to guide surgical nodal staging, a gold standard work-up strategy is needed in order to avoid understaging or overstaging. The aim of this study was to compare diagnostic accuracy and economic impact of three different work-up strategies to identify preoperative risk groups in apparent early-stage EC.

## Materials and methods

### 

This retrospective multicentre study includes all patients with apparent early-stage endometrial cancer diagnosed between September 2016 and December 2018 in three institutions; Regina Montis Regalis Hospital in Mondovì, Policlinico Universitario Gemelli in Rome, and Sacro Cuore Don Calabria Hospital in Negrar. Endometrial cancer diagnosis was based on endometrial biopsy. A patient’s preoperative risk group was identified by the following work-up strategies see Figure1:

**Figure 1:**
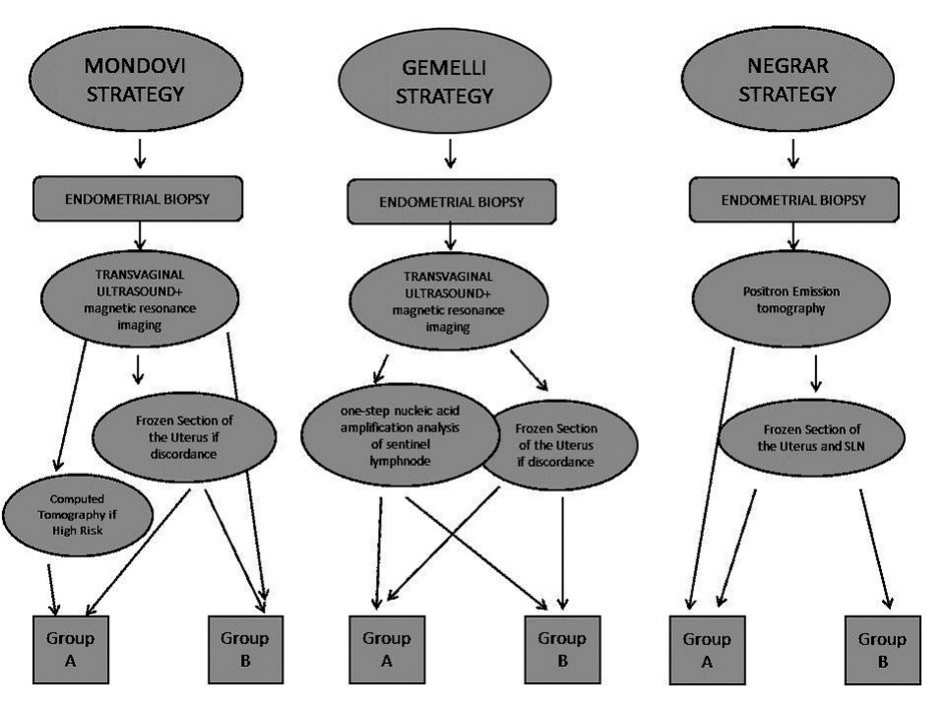


1. - Mondovì Hospital strategy: transvaginal ultrasonography and pelvic MRI for all patients; intraoperative frozen section of the uterus was performed in case of imaging discordance for endometrioid tumours; abdominal CT-scan was requested in high-risk patients.

- Gemelli Hospital strategy: transvaginal ultrasonography, pelvic MRI, and one-step nucleic acid amplification analysis (OSNA) of SLN for all patients; frozen section of the uterus was performed in case of imaging discordance for endometrioid tumours.

- Negrar Hospital strategy: PET and frozen section examination of SLN for all patients; frozen section of the uterus for patients with endometrioid tumours. Endometrium, intrauterine lesions, and myometrial invasion were described according to terms and definitions of the Consensus from the International Endometrial Tumor Analysis Group (Leone et al., 2010). Pelvic MRI was carried out with a 1.5- Tesla system and multi-channels phased-array coil; myometrial invasion was interpreted as superficial or deep (≥50% of myometrium depth) and lymph- nodes were considered pathological if short axis >10 mm (Alcázar et al., 2017).

A CT-scan was performed with and without iodine contrast dye, at 1-2 mm intervals through the pelvis and abdomen during enhancement phases; pelvic and para-aortic lymph nodes were considered enlarged if >15 mm or >10 mm with suspicious features. (Connor et al., 2000).

PET-scan was performed using 18F-fluorodeox- yglucose as per standard clinical protocol; visible lymph nodes were assessed using bidimensional measurements and intensity was assessed using the maximum standardised uptake value (SUV) (Signorelli et al., 2015; Stewart et al., 2019).

All ultrasound, MRI, CT-scans and PET-scans were performed by sonographers and radiologists with at least 2 years’ experience in gynaecological oncology. Frozen sections were used to estimate intraoperatively if tumour myometrial invasion was more or less than 50%. The specimen was intraoperatively delivered to an experienced pathologist, opened along both lateral walls, sliced transversely from the mucosa to the serosa and evaluated both macroscopically and microscopically. Frozen section analysis was compared with the final pathology report (Stephan et al., 2014). All patients included in this study underwent laparoscopic indocyanine-green SLN mapping via cervical injection and SLN biopsy according to the Memorial Sloan-Kettering Cancer Center algorithm (Abu-Rustum et al., 2014; Holloway et al., 2017).

At Gemelli hospital intraoperative OSNA analysis of SLN was routinely performed (Fanfani et al., 2018). The OSNA assay can determine mRNA copy numbers using reverse transcription loop-mediated isothermal amplification (RT– LAMP) reactions. The cut-off of the OSNA method for the description of SLN metastases were less than 160 copies of CK19 mRNA/μL were evaluated as negative, between 160–250 copies of CK19 mRNA/μL as isolated tumour cells (ITC), from 250 to 4999 copies of CK19 mRNA/μL as micrometastases, while more than 5000 copies of CK19 mRNA/μL as macrometastases. At Negrar hospital pathological assessment of SLNs was done initially by intraoperative frozen section evaluation and then with ultrastaging analysis on final pathology. Frozen section of SLNs foresees a multilevel sectioning of the frozen tissue which is subsequently stained with haematoxylin and eosin (H&E) and examined intraoperatively to detect tumoral cells. Mondovì and Negrar hospitals shared the same SLN ultrastaging protocol (Kim et al., 2013; Abu-Rustum et al., 2014) as follows; the initial examination was performed using H&E staining; if the H&E assessment was negative, 2 adjacent 5-μ sections were cut from each paraffin block at each of 2 levels, 50 μ apart. At each level, one side was stained with H&E and the other with immunohistochemistry using anticytokeratin AE1:AE3 (Ventana Medical Systems, Inc., Tucson, AZ) for a total of 4 slides per block.

All patients underwent total laparoscopic hysterectomy with bilateral salpingo-oophorectomy.

For statistical purposes all patients were assigned preoperatively and postoperatively only to two groups, according to the following criteria:

- Group A (high risk): endometrioid EC high risk group (grade 3 with myometrial invasion ≥50%), non-endometriod tumours, International Federation of Gynecology and Obstetrics (FIGO) stage II and suspected stage III.

- Group B (not high risk): endometrioid EC low risk group (grade 1 or 2 with myometrial invasion <50%) and intermediate risk group (grade 1 or 2 with myometrial invasion ≥50%, or grade 3 with myometrial invasion <50%).

Patients were considered misclassified when a switch, from a preoperative group A to a postoperative group B (and vice versa) occurred.

The study was approved by the Ethical Committee (protocol number: ASLCN1/GIN2). Data was collected in an electronic database.

### Statistical analysis

Absolute and percentage frequencies were used to describe patient characteristics. Differences in patients’ preoperative and postoperative characteristics between the three institutions were assessed performing Levene’s Test of Equality of Variances and Anova On Way Test.

Final pathology reports were used to assess the diagnostic accuracy of the three strategies.

The T Student’s test, Wilcoxon rank sum test, Pearson’s Chi-squared test were used to analyse the differences among the three strategies in terms of overall risk group switch, which included work up strategy failures and biopsy errors. Sensitivity, specificity, test accuracy and odds ratio with relevant 95% confidence intervals were calculated for each strategy, excluding biopsy errors that were considered as systematic. RStudio Version 1.2.1335/2009-2019/Rstudio, Inc. was used for statistical analysis and a p-value 0.05 was considered statistically significant.

### Cost estimate

Work-up strategy costs were estimated, at patient level, as the sum of all examination costs. Diagnostic test costs were estimated using reimbursement tariffs (in Euros, €) as rewarded to suppliers by the Italian National Health System. The cost of the intraoperative frozen-section examination includes both the pathological analysis and the extra operating-room time, valued as hourly costs provided by the hospital administration. Regarding the two preoperative groups of this study (preoperative group A and group B), costs were described as patient’s mean cost of each work-up strategy. A Pearson’s Chi-squared test was used to assess the difference of patient’s mean cost among the three institutions and a p-value 0.05 was considered statistically significant.

## Discussion

Three hundred eighty-five patients were included in the study; 93 at Mondovì Hospital, 215 at Gemelli Hospital and 77 at Negrar Hospital. Table I summarises pre- and intraoperative clinicopathologic data with no significant difference among the three centres. Ten (10.7%) patients at Mondovì Hospital and 17 (7.9%) patients at Gemelli Hospital did not undergo MRI because of implants, cardiac pacemaker, or claustrophobia. The CT-scans, performed in 18 (19.4%) preoperatively high risk patients at Mondovì hospital, showed a suspiciously enlarged lymph node in one case, which was found to be metastatic at the final pathology result. The PET-scan, performed in all Negrar patients, suspected extra-uterine disease in 8 (10.4%) cases: two were false-positive (one suspicious for lymph node involvement and one for parametrial involvement). Frozen section of the uterus assessing myometrial invasion was requested in 20 (21.5%), 38 (17.7%) and 63 (81.8%) patients in Mondovì, Gemelli and Negrar Hospital respectively. The final pathology report of the uterus was always concordant with the intraoperative frozen section analysis in all institutions, except for two frozen section errors at Negrar Hospital. Frozen section of SLN was performed in 56 (72.7%) patients at Negrar; one SLN macrometastasis was missed at frozen section and then detected at final pathology. OSNA of SLN, performed for all Gemelli patients, revealed 33 SLN metastasis (5 macrometastasis, 24 micrometastasis and 4 ITC) intraoperatively.

**Table I:** Patients’ Preoperative and intraoperative characteristics of the patients at the three hospitals .

	Mondovì Hospital	Gemelli Hospital	Negrar Hospital	Total	
	(N = 93)	(N=215)	(N = 77)	(N = 385)	p-value
Age					
Mean (SD)	66.25 (10.44)	59.94 (10,73)	64.68 (9.79)	62.46 (10.93)	0.7421
Menopausal status					
No	5 (5.4%)	46 (21.4%)	9 (11.7%)	60 (15.5%)	0.5746
Yes	88 (94.6%)	169 (78.6%)	68 (88.3%)	326 (84.5%)	
Body Mass Index					
Mean (SD)	28.13 (5.61)	28.79 (6.51)	28.55 (6.41)	28.00 (6.27)	0.1918
Preoperative histology					0.7269
Endometrioid	85 (91.3%)	204 (94.9%)	69 (89.6%)	358 (92.7%)	
Carcinosarcoma	2 (2.2%)	2 (0.9%)	1 (1.3%)	5 (1.3%)	
Serous	4 (4.3%)	7 (3.3%)	5 (6.5%)	16 (4.1%)	
Clear Cells	0 (0.0%)	2 (0.9%)	2 (2.6%)	4 (1.0%)	
Undifferentiated	2 (2.2%)	0 (0.0%)	0 (0.0%)	2 (0.5%)	
Preoperative grading*					0.6741
Grade 1	37 (43.5%)	87 (42.6%)	53 (76.8%)	177 (49.4%)	
Grade 2	33 (38.8%)	98 (48.0%)	8 (11.6%)	139 (38.8%)	
Grade 3	15 (17.6%)	19 (9.3%)	8 (11.6%)	42 (11.7%)	
Transvaginal Ultrasound assessing myometrial invasion					
<50%	40 (43.0%)	144 (67.3%)		184 (59.9%)	
≥50%	53 (57.0%)	70 (32.7%)		123 (40.1%)	
MRI assessing myometrial invasion					
<50%	31 (37.3%)	138 (69.6%)		169 (60.1%)	
≥50%	52 (62.7%)	60 (30.3%)		112 (39.9%)	
PET assessing extra-uterine disease					
Absent			69 (89.6%)		
Present			8 (10.4%)		
CT assessing extra-uterine disease**					
Absent	17 (98.9%)				
Present	1 (1,1%)				
Frozen-section assessing myometrial invasion					
<50%	9 (45.0%)	26 (79.6%)	50 (79.4%)	53 (77.9%)	
>50%	11 (55.0%)	12 (20.4%)	13 (20.6%)	15 (22.1%)	
Frozen-section of SLN					
Negative			55 (98,2%)		
Positive			1 (1,8%)		
OSNA of SLN					
Negative			185 (86.0%)		
Positive			30 (14.0%)		

MRI: Magnetic resonance imaging; PET: positron emission tomography scan; CT: abdominal computed tomography; SLN: sentinel lymph node; OSNA: One Step Nucleic Acid Amplification; * endometrioid cancer; ** patients preoperatively classified as high-risk.

**Table II:** Summary of postoperative patient characteristics. FIGO stage IA was reported in 44.1% at Mondovì Hospital, in 62.3% at Gemelli and in 74.0% cases at Negrar; only 1 patient with FIGO stage IIIC1 (1.3%) was found at Negrar, versus 15 (16.1%) and 25 (11.6%) at Mondovì and Gemelli hospital, respectively. Patients correctly classified and those misclassified because of risk group switch (from group A to group B and vice versa) for each hospital are reported in Table III.

	Mondovì Hospital	Gemelli Hospital	Negrar Hospital	Total	
	(N = 93)	(N=215)	(N = 77)	(N = 385)	p-value
Postoperative histology					0.7133
Endometrioid	82 (88.2%)	197 (91.6%)	70 (90.9%)	349 (90.%)	
Carcinosarcoma	5 (5.3%)	1 (0.5%)	1 (1.3%)	7 (1.8%)	
Serous	4 (4.3%)	12 (5.6%)	4 (5.2%)	20 (5.2%)	
Clear Cells	0 (0.0%)	3 (1.4%)	2 (2.6%)	5 (1.3%)	
Undifferentiated	2 (2.2%)	2 (0.9%)	0 (0.0%)	4 (1.0%)	
Postoperative grading*					0.631
Grade 1	33 (40.2%)	30 (15.2%)	51 (71.8%)	114 (32.65%)	
Grade 2	32 (39.1%)	138 (70.1%)	13 (18.3%)	183 (52.3%)	
Grade 3	17 (20.7%)	29 (14.7%)	7 (9.9%)	53 (15.1%)	
Postoperative FIGO stage					0.3323
I A	41 (44.1%)	134 (62.3%)	57 (74.0%)	232 (60.1%)	
I B	25 (26.9%)	43 (20.0%)	15 (19.5%)	84 (21.8%)	
II	5 (5.4%)	8 (3.7%)	1 (1.3%)	14 (3.6%)	
III A	3 (3.2%)	2 (0.9%)	1 (1.3%)	6 (1.6%)	
III B	2 (2.2%)	0 (0.0%)	0 (0.0%)	2 (0.5%)	
III C1	15 (16.1%)	25 (11.6%)	1 (1.3%)	41 (10.6%)	
III C2	2 (2.1%)	3 (1.4%)	2 (2.6%)	7 (2.8%)	
Myometrial invasion					0.3241(a)
<50%	45 (48.4%)	154 (71.6%)	58 (75.3%)	257 (66.8%)	
≥50%	48 (51.6%)	61 (28.4%)	19 (24.7%)	129 (33.2%)	
Lymphovascular space invasion					0.3757(a)
Negative	72 (77.4%)	151 (70.2%)	62 (80.5%)	286 (74.1%)	
Positive	21 (22.6%)	64 (29.8%)	15 (19.5%)	100 (25.9%)	
SLN number					0.5996(a)
Mean (SD)	2.25 (0.985)	1.98 (0.63)	2.36 (1.74)	2.12(1.15)	
Metastastatic SLN					0.7775(a)
No	74 (83.1%)	184 (85.6%)	65 (94.2%)	323 (86.6%)	
Yes	15 (16.9%)	31 (14.4%)	4 (5.8%)	50 (13.4%)	
SLN Macrometastasis					0.1506(a)
Absent	9 (64.3%)	3 (37.5%)	2 (50.0%)	14 (53.8%)	
Present	5 (35.7%)	5 (62.5%)	2 (50.0%)	12 (46.2%)	
SLN Micrometastasis					0.6527(a)
Absent	9 (64.3%)	0 (0.0%)	4 (100.0%)	13 (31.0%)	
Present	5 (35.7%)	24 (100%)	0 (0.0%)	29 (69.0%)	
SLN Isolated Tumor Cells					0.2677(a)
Absent	10 (71.4%)	4 (50.0%)	2 (50.0%)	16 (61.5%)	
Present	4 (28.6%)	4 (50.0%)	2 (50.0%)	10 (38.5%)	
Nodal surgical staging					0.688
Only SLN	42 (45.2%)	155 (72.1%)	49 (63.6%)	246 (63.9%)	
SLN+ pelvic lymphadenectomy	37 (39.8%)	39 (18.1%)	18 (23.4%)	94 (24.4%)	
SLN+ pelvic and para-aortic lymphadenectomy	14 (15.1%)	21 (9.8%)	10 (13.0%)	45 (11.7%)	
Pelvic no-SLN metastasis					0.8571(a)
No	45 (80,6%)	45 (84.9%)	27 (96.4%)	117 (88.6%)	
Yes	6 (19,4%)	8 (15.1%)	1 (3.6%)	15 (11.4%)	
Para-aortic no-SLN metastasis					0.8288(a)
No	12 (85.7%)	16 (84.2%)	8 (80.0%)	36 (83.7%)	
Yes	2 (14.3%)	3 (15.8%)	2 (20.0%)	7 (16.3%)	

FIGO: International Federation of Gynecology and Obstetrics; SLN: sentinel lymph node; * endometrioid cancer; (a) ANOVA one-way test

**Table III:** Patients correctly classified and patients misclassified.

	Mondovì Hospital	Gemelli Hospital	Negrar Hospital
Patients correctly classified	73 (78.49%)	188 (87.44%)	69 (89.61%)
Risk group switch: group A → group B	1 (1.08%)	0 (0.00%)	4 (5.19%)
Risk group switch: group B → group A	14 (15.05%)	10 (4.65%)	0 (0.00%)
Risk group switch due to biopsy error	5 (5.38%)	17 (7.91%)	4 (5.19%)

Preoperative endometrial biopsy failures were responsible for 47.3% of all misclassifications. Diagnostic accuracy of the three work-up strategies was not statistically different when biopsy errors were included in the risk group rate switch. However, since biopsy error was considered a systematic uneditable error, cases of switching due to endometrial biopsy were excluded to calculate sensitivity and specificity, for each work-up strategy, with the following results: 56.2% and 98.2% for Mondovì strategy, 81.5% and 100% for Gemelli, and 100% and 93.6% for Negrar.

The test accuracy of Mondovì, Gemelli and Negrar work-up strategies was 0.83 (95%CI 0.734-0.901), 0.95 (95%CI 0.909-0.975) and 0.94 (95%CI 0.866- 0.985), respectively.

When Odd-Ratio was calculated, Negrar and Gemelli strategies appeared to be more effective compared to Mondovì one: Odd-Ratio was 0.28 (95%CI 0.065-0.950) for Mondovì versus Negrar strategy, and 0.26 (95%CI 0.099-0.651) for Mondovì versus Gemelli strategy. No significant differences between the Gemelli and Negrar strategies were noted: Odd-Ratio 0.92 (95%CI 0.254-4.142). Among the 15 misclassified patients by Mondovì hospital strategy, there was one failure assessing myometrial invasion by concordant transvaginal ultrasound and MRI imaging. Three cases of cervical involvement (FIGO stage II) remained undetected by MRI; 2 of them because of microscopic stromal invasion. Eleven patients with extra-uterine disease (FIGO stage III) were noted: 4 with nodal macrometastasis and 3 with nodal micrometastasis (FIGO stage IIIC), 3 cases of FIGO stage IIIA (focal and millimetric lesions of the adnexa or the perimetrium), and one case of FIGO stage IIIB (millimetric invasion of parametria).

Among the 10 misclassified patients by the Gemelli strategy there were as follows: one failure assessing myometrial invasion by concordant MRI and transvaginal ultrasound, 6 cases of microscopic cervical stromal invasion (FIGO stage II) and 3 patients with non-SLN nodal macrometastasis (FIGO stage IIIC). Among the 4 misclassified patients by the Negrar strategy, there were two cases of false-positive PET scan exams (one case showing nodal metastasis and one for parametrial involvement), and two cases of frozen section failures assessing myometrial invasion.

The mean cost per patient of preoperative work-up in group A at Mondovì, Gemelli, and Negrar Hospitals was €514.50 ($556.80), €868.50 ($939.90) and €1212.80 ($1312.50), respectively (p-value < 0.001). The mean cost per patient of preoperative work-up in group B at Mondovì, Gemelli, and Negrar Hospitals was €378.80 ($409.90), €941.20 ($1018.60) and €1848.40 ($2000.40), respectively (p-value < 0.001) (Figure 2).

**Figure 1:**
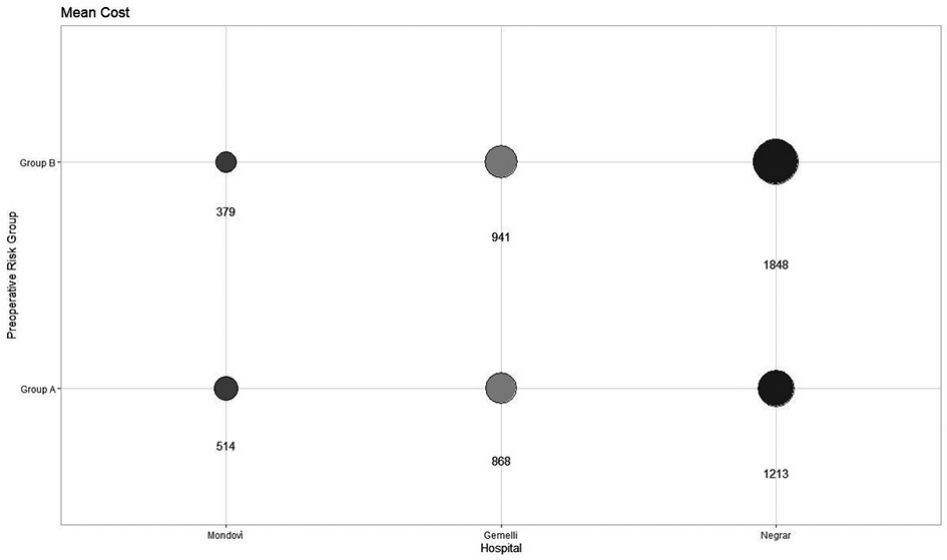
The mean cost per patient of preoperative work-up in Mondovi, Gemelli and Negrar Hospitals.

## Discussion

In this study Negrar and Gemelli strategies had similar diagnostic accuracy and were proven to be more accurate than Mondovì one. The Negrar strategy was the most expensive, while the most economical was at Mondovì. Since clinicians must rely on work-up strategies, which are a planned sequence of examinations, the accuracy of real-life work-up strategies was the focus of our study. We also consider the economic impact a relevant issue providing insights on health system sustainability and patient care effectiveness. In fact, Italian public Hospitals have to follow the rules of the Regional Health Services: for instance, the Piedmont Oncology Network does not provide routinely PET-scan for endometrial cancer staging purposes (Rete Oncologica Piemonte Valle d’Aosta, www.reteoncologica.it). Since in EC small-volume (tumour size < 2 cm) low risk patients incidences of nodal involvement are detected around 1%, while in high risk patients it is around 20-40% (Mariani et al., 2008; Kumar et al,. 2014), an accurate preoperative identification of risk groups is essential to select patients who can truly benefit from lymphadenectomy.

However, several work-up tool options are described in the ESMO/ESGO/ESTRO consensus conference, allowing hospitals to adopt different work-up strategies (Colombo et al., 2016) which lead to different diagnostic accuracy and economic impact. Preoperative tools included in the three work-up strategies of this study showed their strengths and limitations. Regarding preoperative endometrial biopsy errors, our data are in line with those previously published showing a modest predictive value for postoperative histological grading with an overall concordance of 60.75% (Batista et al., 2016). In order to improve the quality of endometrial biopsy, some authors suggest using a binary scheme to grade endometrioid tumours, considering grade I and II tumours as “low-grade” and grade III tumors as “high-grade” and to incorporate the four genomic endometrial carcinoma categories (Soslow et al., 2019). To assess myometrial, cervical and extra-uterine disease, even expensive exams such as PET-scan, CT-scan and frozen section do not reach 100% accuracy (Stephan et al., 2014; De Bernardi et al., 2018). Transvaginal ultrasound and MRI detection rates in our series are similar to those reported in the literature (Fischerova et al., 2014; Alcázar et al., 2017; Brocker et al., 2019). Our results highlight one of the main limitations of work-up imaging; the microscopic invasion of parametria, cervix or adnexae reported at final pathology, and the microscopic tumour invasion causing risk group switch to FIGO stage II or III and affecting adjuvant treatment- no validated imaging is currently available to overcome this issue.

Misclassified patients due to nodal macrometastasis >5mm could have probably benefited from a PET-scan, while incorrect myometrial invasion assessment due to a concordant but incorrect transvaginal ultrasound and MRI, might have been avoided with frozen section analysis. Regarding work-up tool strengths, frozen section was the only exam which revealed one case of cervical invasion (FIGO stage II), the PET-scan showed suspicious nodal metastasis (confirmed at final pathology) in two patients, and OSNA of SLN uncovered intraoperatively metastasis in 30 patients. This study provides an opportunity for an open discussion on different SLN analysis techniques; while SLN intraoperative ultrastaging is not feasible, SLN frozen section could potentially alter the node for final ultrastaging (Holloway et al., 2017). OSNA analysis showed high sensitivity and specificity to detect SLN metastasis intraoperatively, including low volume metastasis (Kosťun et al., 2018); however false-positive OSNA results due to the presence of benign glandular epithelial inclusions can be a challenging issue, even if the rate of this finding is very low (Fanfani et al., 2018). OSNA of SLN could be an upgrade of work-up strategies in EC, but, currently, its role is limited due to high costs and availability issues in Italian oncological centres.

According to ESMO/ESGO/ESTRO recommendations, lymphadenectomy is not recommended for low risk patients and it can be considered for staging purpose for intermediate risk patients; while high risk patients are eligible for systematic pelvic and para-aortic lymphadenectomy (Colombo et al., 2016; 2017). At the same time, the systematic pelvic and para-aortic lymphadenectomy for high risk patients are no longer considered as a treatment option , due to recent studies showing no statistical difference in survival when comparing the SLN algorithm with systematic lymphadenectomy (Buda et al., 2018; Schlappe et al., 2018;, Schlappe et al., 2020).

Taking into account that the risk of isolated para- aortic metastasis is only about 1-3% (Kumar et al., 2014) and since SLN algorithm for nodal staging was performed in all patients, it was considered just a partial understaging when systematic lymphadenectomy was not performed in high risk patients of this study. On the other hand, we consider overstaging a major issue because performing an unnecessary lymphadenectomy can cause severe surgical complications (Dowdy et al., 2012; Yost et al., 2014) in older, overweight and multi-morbid patients (Boll et al., 2011; Arem et al., 2013). Many studies have been published investigating the diagnostic accuracy of single preoperative imaging in EC but, to our knowledge, this is the first study reporting the accuracy of a whole work-up strategy identifying EC risk groups accompanied by cost- analysis. Homogeneous protocols in terms of laparoscopic surgery and SLN surgical technique are one of the main strengths of this study. Limitations of the study are the retrospective design and the slightly different distribution (not statistically different) of postoperative FIGO stage in the three hospitals.

The FIRES trial showed that SLN mapping with indocyanine-green has a high degree of diagnostic accuracy in detecting endometrial cancer metastases and can safely replace lymphadenectomy in the staging of EC (Rossi et al., 2017). Although the SLN algorithm is gaining widespread acceptance among gynaecological oncologists worldwide (Casarin et al., 2019), its application is still an optional procedure according to the most recent European and American guidelines (Colombo et al., 2016; NCCN 2020). Therefore, performing an accurate preoperative identification of risk groups is still of paramount importance to direct surgical nodal staging and to avoid under- or overstaging. Because of the intrinsic limits of work-up tools, we suggest that future guidelines should clarify which work-up strategy should be the gold standard to be offered in every oncological centre, as well as focusing on the role of SLN and its most appropriate examination technique.

## References

[1] Abu-Rustum NR (2014). Sentinel lymph node mapping for endometrial cancer: a modern approach to surgical staging.. J Natl Compr Canc Netw.

[2] Alcázar JL, Gastón B, Navarro B (2017). Transvaginal ultrasound versus magnetic resonance imaging for preoperative assessment of myometrial infiltration in patients with endometrial cancer: a systematic review and meta-analysis.. J Gynecol Oncol.

[3] Arem H, Irwin ML (2013). Obesity and endometrial cancer survival: a systematic review.. International journal of obesity (2005).

[4] Ballester M, Dubernard G, Lecuru F (2011). Detection rate and diagnostic accuracy of sentinel-node biopsy in early stage endometrial cancer: a prospective multicentre study (SENTI-ENDO).. Lancet Oncol.

[5] Batista TP, Cavalcanti CL, Tejo AA (2016). Accuracy of preoperative endometrial sampling diagnosis for predicting the final pathology grading in uterine endometrioid carcinoma.. Eur J Surg Oncol.

[6] Benedetti Panici P, Basile S, Maneschi F (2008). Systematic pelvic lymphadenectomy vs. no lymphadenectomy in early-stage endometrial carcinoma: randomized clinical trial. J Natl Cancer Inst.

[7] Bogani G, Dowdy SC, Cliby WA (2014). Role of pelvic and para-aortic lymphadenectomy in endometrial cancer: current evidence.. J Obstet Gynaecol Res.

[8] Boll D, Verhoeven RH, Van der Aa MA (2011). Adherence to national guidelines for treatment and outcome of endometrial cancer stage I in relation to co-morbidity in southern Netherlands 1995–2008.. Eur J Cancer.

[9] Brocker KA, Radtke JP, Hallscheidt P (2019). Comparison of the determination of the local tumor extent of primary endometrial cancer using clinical examination and 3 Tesla magnetic resonance imaging compared to histopathology.. Arch Gynecol Obstet.

[10] Buda A, Gasparri ML, Puppo A (2018). Lymph node evaluation in high-risk early stage endometrial cancer: A multi-institutional retrospective analysis comparing the sentinel lymph node (SLN) algorithm and SLN with selective lymphadenectomy.. Gynecol Oncol.

[11] Buda A, Restaino S, Di Martino G (2018). The impact of the type of nodal assessment on prognosis in patients with high-intermediate and high-risk ESMO/ESGO/ESTRO group endometrial cancer. A multicenter Italian study.. Eur J Surg Oncol.

[12] Casarin J, Multinu F, Abu-Rustum N (2019). Factors influencing the adoption of the sentinel lymph node technique for endometrial cancer staging: an international survey of gynecologic oncologists.. Int J Gynecol Cancer.

[13] Colombo N, Creutzberg C, Amant F (2016). ESMO-ESGO-ESTRO consensus conference on endometrial cancer: diagnosis, treatment and follow-up.. Ann Oncol.

[14] Colombo N, Creutzberg C, Querleu D (2017). Appendix 5: Endometrial cancer. Ann Oncol.

[15] Connor JP, Andrews JI, Anderson B (2000). Computed tomography in endometrial carcinoma.. Obstet Gynecol.

[16] De Bernardi E, Buda A, Guerra L (2018). Radiomics of the primary tumour as a tool to improve 18F-FDG-PET sensitivity in detecting nodal metastases in endometrial cancer.. EJNMMI Res.

[17] Dowdy SC, Borah BJ, Bakkum-Gamez JN (2012). Prospective assessment of survival, morbidity, and cost associated with lymphadenectomy in low-risk endometrial cancer.. Gynecol Oncol.

[18] Ehrisman J, Secord AA, Berchuck A (2016). Performance of sentinel lymph node biopsy in high-risk endometrial cancer.. Gynecol Oncol Rep.

[19] Fanfani F, Monterossi G, Ghizzoni V (2018). One-step nucleic acid amplification (OSNA): A fast molecular test based on CK19 mRNA concentration for assessment of lymph-nodes metastases in early stage endometrial cancer.. PLoS One.

[20] Fischerova D, Fruhauf F, Zikan M (2014). Factors affecting sonographic preoperative local staging of endometrial cancer.. Ultrasound Obstet Gynecol.

[21] Holloway RW, Abu-Rustum NR, Backes FJ (2017). Sentinel lymph node mapping and staging in endometrial cancer: a society of gynecologic oncology literature review with consensus recommendations.. Gynecol Oncol.

[22] Kim CH, Soslow RA, Park KJ (2013). Pathologic ultrastaging improves micrometastases detection in sentinel lymph nodes during endometrial cancer staging.. Int J Gynecol Cancer.

[23] Kitchener H, Swart AM, Qian Q (2009). Efficacy of systemic pelvic lymphadenectomy in endometrial cancer (MRC ASTEC trial): a randomised study.. Lancet.

[24] Kosťun J, Pešta M, Sláma J (2019). One-step nucleic acid amplification vs ultrastaging in the detection of sentinel lymph node metastasis in endometrial cancer patients.. J Surg Oncol.

[25] Kumar S, Podratz KC, Bakkum-Gamez JN (2014). Prospective assessment of the prevalence of pelvic, paraaortic and high paraaortic lymph node metastasis in endometrial cancer. Gynecol Oncol.

[26] Leone FP, Timmerman D, Bourne T (2010). Terms, definitions and measurements to describe the sonographic features of the endometrium and intrauterine lesions: a consensus opinion from the International Endometrial Tumor Analysis (IETA) group.. Ultrasound Obstet Gynecol.

[27] Mariani A, Dowdy SC, Cliby WA (2008). Prospective assessment of lymphatic dissemination in endometrial cancer: a paradigm shift in surgical staging.. Gynecol Oncol.

[28] (2020). NCCN guidelines. Uterine Neoplasms. Version 1. Version 1.

[29] Piedmont Oncology Network website.

[30] Rossi EC, Kowalski LD, Scalici J (2017). A comparison of sentinel lymph node biopsy to lymphadenectomy for endometrial cancer staging (FIRES trial): a multicentre, prospective, cohort study.. Lancet Oncol.

[31] Schlappe BA, Weaver AL, Ducie JA (2018). Multicenter study comparing oncologic outcomes between two nodal assessment methods in patients with deeply invasive endometrioid endometrial carcinoma: A sentinel lymph node algorithm versus a comprehensive pelvic and paraaortic lymphadenectomy.. Gynecol Oncol.

[32] Schlappe BA, Weaver AL, McGree ME (2020). Multicenter study comparing oncologic outcomes after lymph node assessment via a sentinel lymph node algorithm versus comprehensive pelvic and paraaortic lymphadenectomy in patients with serous and clear cell endometrial carcinoma.. Gynecol Oncol.

[33] Signorelli M, Crivellaro C, Buda A (2015). Staging of high-risk endometrial cancer with PET/CT and sentinel lymph node mapping.. Clin Nucl Med.

[34] Soliman PT, Westin SN, Dioun S (2017). A prospective validation study of sentinel lymph node mapping for high-risk endometrial cancer.A prospective validation study of sentinel lymph node mapping for high-risk endometrial cancer.. Gynecol Oncol.

[35] Soslow RA, Tornos C, Park KJ (2019). Endometrial Carcinoma Diagnosis: Use of FIGO Grading and Genomic Subcategories in Clinical Practice: Recommendations of the International Society of Gynecological Pathologists.. Int J Gynecol Pathol.

[36] Stephan JM, Hansen J, Samuelson M (2014). Intra-operative frozen section results reliably predict final pathology in endometrial cancer.. Gynecol Oncol.

[37] Stewart KI, Chasen B, Erwin W (2019). Preoperative PET/CT does not accurately detect extrauterine disease in patients with newly diagnosed high-risk endometrial cancer: A prospective study.. Cancer.

[38] Todo Y, Kato H, Kaneuchi M (2010). Survival effect of para-aortic lymphadenectomy in endometrial cancer (SEPAL study): a retrospective cohort analysis.. Lancet.

[39] Touhami O, Grégoire J, Renaud MC (2017). Performance of sentinel lymph node (SLN) mapping in high-risk endometrial cancer.. Gynecol Oncol.

[40] Yost KJ, Cheville AL, AlHilli MM (2014). Lymphedema after surgery for endometrial cancer: prevalence, risk factors, and quality of life.. Obstet Gynecol.

